# Clozapine-Related Diarrhea and Colitis: Report of 4 Cases

**DOI:** 10.1097/JCP.0000000000001204

**Published:** 2020-04-27

**Authors:** Susanna Maria Rask, Kaisa E. Luoto, Anssi Solismaa, Elina Jokinen, Airi Jussila, Olli Kampman

**Affiliations:** From the ∗ Department of Psychiatry, Faculty of Medicine and Health Technology, Tampere University; † Department of Psychiatry, Tampere University Hospital, Pirkanmaa Hospital District; ‡ Psychiatric Unit, Mental Health and Substance Abuse Services, City of Tampere; § Department of Gastroenterology and Alimentary Tract Surgery, Tampere University Hospital, Pirkanmaa Hospital District, Tampere, Finland.

**Keywords:** clozapine, diarrhea, eosinophilic enteropathy, microscopic colitis, Charcot-Leyden crystal

## Abstract

**Background:**

During clozapine treatment, diarrhea is a rare but clinically relevant adverse effect. Cases of microscopic colitis and eosinophilic colitis have been previously reported.

**Procedures:**

We present 4 patients who developed severe diarrhea in early weeks of clozapine therapy.

**Findings:**

Two patients had significant peripheral eosinophilia 1 week after diarrhea symptoms. One of these patients also had Charcot-Leyden crystals in stool afterward, confirming the presence of eosinophils in the gut lumen. One of our patients had a confirmed microscopic colitis and later also neutropenia, which required treatment.

**Conclusions:**

Charcot-Leyden crystals in stool may be associated with concurrent diarrhea and eosinophilia during clozapine treatment, which is a previously unreported finding. Occurrence of blood dyscrasias with diarrhea symptoms during clozapine treatment needs further investigation to understand the possible shared mechanisms.

During clozapine treatment, diarrhea and colitis have been reported as rare adverse effects,^1^ and the association might not be well recognized among professionals. The mechanisms behind clozapine-related colitis remain unclear, and it seems that there might be various associative etiological agents, such as eosinophilic colitis. In recent years, a growing body of case reports is suggesting that microscopic colitis may be a complication of clozapine therapy.^1,2^ Interestingly, there were 4 patients within 1 year in the psychiatric unit of Tampere University Hospital who presented diarrhea after commencement of clozapine treatment. In this article, we report these cases and discuss their symptoms in relation to previously reported cases.

## PATIENTS

All 4 patients were treated during January 2018 to January 2019 as inpatients in the psychiatric unit of Tampere University Hospital in Finland. The hospital belongs to Pirkanmaa Hospital District, and the psychiatric unit provides specialist care for 530,000 inhabitants. The patients were white, followed a normal mixed food diet, and had not been traveling abroad or used antibiotics preceding the admission. The patients did not have any diagnosed long term somatic medical conditions. None of the patients had any known family history of inflammatory bowel diseases or celiac disease. Other patients in the same wards did not suffer from any gastrointestinal symptoms at the same time, and thus there was no sign of infectious gastroenteritis spreading in the ward. The clozapine came from a different medicine lot for every patient. Table 1 shows the patients' allergies, smoking status, and medications before initiation of clozapine, and Table 2 summarizes the changes in the leukocyte, neutrophil, and eosinophil counts during the clozapine treatment attempts for each patient. The study was performed as a registry study based solely on the patient registry data, which in Finland requires no informed consent, and in accordance to the ethical code of Tampere University Hospital.

**TABLE 1:**
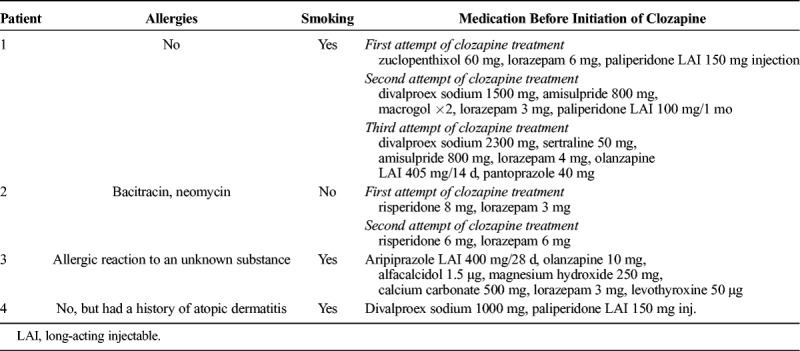
Patients' Allergies, Regular Smoking Status, and Medications Before Initiation of Clozapine

**TABLE 2:**
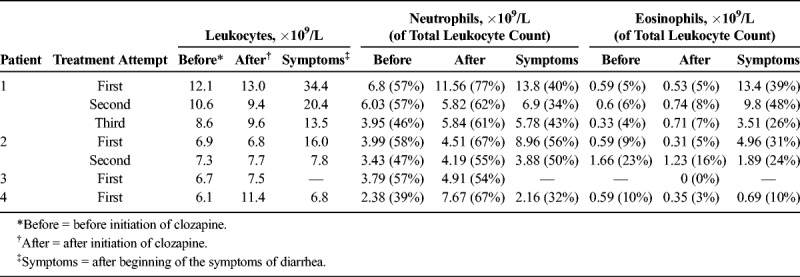
Leukocyte, Neutrophil, and Eosinophil Counts Before and After Clozapine Initiation and After the Development of Diarrhea

Patient 1 is a 38-year-old man with previous episodes of treatment-refractory schizophrenia. After 2 weeks of clozapine treatment as the dose reached 200 mg, the patient had fever, nausea, vomiting, and watery diarrhea, and he was treated with antibiotics (ceftriaxone, metronidazole) for a week. Infectious diseases were ruled out, as was celiac disease. One week after the onset of diarrhea, the patient started to develop eosinophilia. The bone marrow biopsy was taken showing abundant eosinophilia, but no sign of hematological disease was found. Antibiotic treatment was discontinued, and prednisolone was commenced. Clozapine treatment was also discontinued, the gastrointestinal symptoms abated, and eosinophil count returned to normal. Three months after the acute diarrhea phase, gastroscopy and colonoscopy were done, and all the biopsy findings were normal, although the patient had signs of diverticulosis in sigma. After the colonoscopy, clozapine was restarted. This led to reemergence of diarrhea symptoms, although milder than on first attempt. Eosinophil levels rose rapidly and remained high (Table 2). Fecal parasites were tested and were negative. Clozapine was then again discontinued, and eosinophilia and gastrointestinal symptoms disappeared. Because of extreme severity of his psychiatric symptoms, clozapine was started again for the third time 10 months after the second attempt. This time the level of eosinophils rose in 3 weeks after commencement and then dropped gradually, reaching normal levels in 3 months. Further gastrointestinal symptoms or any other major complications have not been reported.

Patient 2 is a 38-year-old man with a first hospitalization due to schizophrenia symptoms. At 2 weeks after the commencement of clozapine (dose 125 mg), the patient started to have flulike symptoms and diarrhea. Infectious etiology was ruled out, as was celiac disease. Eosinophilia developed 1 week later, and clozapine was discontinued. The symptoms of diarrhea subsided. Nine weeks after the discontinuation of clozapine, the stool sample showed Charcot-Leyden crystals (CLCs), that is, eosinophilic breakdown products that are usually found in stool in conjunction with parasitic infections. Also, this patient was rechallenged with clozapine treatment. Sigmoidoscopy was performed during the rechallenge (clozapine dose 75 mg), and biopsies showed normal histology. The level of eosinophils again rose after 1 week of new onset of treatment, and as the clozapine dose reached 175 mg/d, patient started having watery diarrhea several times a day. This time the number of peripheral eosinophils was lower when compared with previous blood counts few days earlier. Clozapine was again discontinued, and patient's symptoms of diarrhea disappeared. Parasitic infections were ruled out by several fecal parasitic samples after the second clozapine challenge.

Patient 3 is a 30-year-old woman with a first hospitalization due to psychotic symptoms. During the first week of clozapine treatment (dose 125 mg), she started to have watery diarrhea that led to weight loss. In the laboratory tests, C-reactive protein remained relatively low at 54 mg/L (reference, <4 mg/L), fecal calprotectin that measures intestinal inflammation was 375 μg/g (reference range, 0–100 μg/g), cytomegaloviral antibodies, and celiac disease antibodies were negative. No eosinophilia was detected in this patient. Clozapine was continued at 200 mg during the symptoms. The patient's somatic condition then required an acute colonoscopy, which confirmed a microscopic lymphocytic colitis, and she was treated successfully with budesonide. Neutropenia developed during the third week after commencement of clozapine and was severe with a risk of agranulocytosis, which led to the discontinuation of clozapine and treatment with hematopoietic growth factor and cefuroxime.

Patient 4 is a 23-year-old man with second hospitalization due to psychotic symptoms. Two weeks after the commencement of clozapine (dose 150 mg), the patient started to have watery diarrhea. In the laboratory tests, no eosinophilia was detected for this patient. Fecal calprotectin was elevated at 4112 μg/g, and C-reactive protein was 102 mg/L at its peak. Celiac disease antibodies and cytomegalovirus antibodies were negative, as well as all bacterial and viral and parasitic samples from stool. The patient declined acute colonoscopy. Clozapine was discontinued because of the suspected clozapine related colitis with severe symptoms, and after that, his diarrhea symptoms disappeared without any other treatment. The patient had slightly elevated level of eosinophils in peripheral blood before commencement of clozapine and again after clozapine was discontinued and diarrhea symptoms had abated.

## DISCUSSION

Diarrhea as an adverse effect of clozapine treatment is a rare occurrence. Several case reports have been published, but the pathomechanisms are still speculative. This study presents 4 new cases of clozapine-related diarrhea. The time frame for onset of diarrhea was similar in all our cases as the symptoms began 7 to 14 days after the initiation of clozapine. The relation between diarrhea and clozapine treatment seemed to be quite strong, and in 2 cases, it was seen also after a second attempt of clozapine treatment. However, the possible etiological factors are varying and speculative.

The first reports of clozapine-related diarrheas have been published in 1990s. Harvey et al^3^ wrote about 3 cases of severe diarrhea during clozapine treatment and speculated the coinciding changes in the blood count as a possible related factor. Verbeeck and Berk^4^ reported a case of clozapine-related neutropenia, which was then complicated by cytomegalovirus colitis. Linsley and Williams^1^ summarized the previous literature of clozapine-associated colitis until 2012 and reported a case of a likely pseudomembranous colitis during clozapine treatment. In their review, they included case reports of several types of colitis (2 cases of necrotizing, 2 cases of pseudomembranous, 1 microscopic, 1 acute, and 1 eosinophilic).^5–10^ De Raad et al^2^ reported a case of eosinophilic colitis 2 weeks after commencement of clozapine treatment. More recently, Holz et al^11^ reported a case of microscopic colitis associated with clozapine use and biopsy-proven celiac disease, and Chu and Liang^12^ presented a case with clozapine-associated systemic infection and cytomegalovirus colitis with neither neutropenia nor agranulocytosis. Finnish Medicines Agency^13^ confirmed to have received 1 notification in 2014 of a case with eosinophilia and diarrhea associated with commencement of clozapine. This case was very similar to our cases, as the patient started having diarrhea during early stages of clozapine treatment and then developed eosinophilia. Also, 1 case of ischemic colitis during clozapine treatment has been reported to Finnish Medicines Agency. In the European Medicines Agency's database EudraVigilance,^14^ approximately 120 notes of clozapine-related colitis and approximately 15 notes of eosinophilic colitis can be found. However, causal relationship cannot be deduced from the European Medicines Agency database because confounding factors may not be excluded.

Clozapine treatment is known to be associated with multiple hematologic adverse effects, with agranulocytosis being the most severe and well-known, but also cases of eosinophilia have been reported.^15–17^ An Italian study of clozapine-treated patients (n = 2404) reported a 2.2% incidence of eosinophilia with the criteria of eosinophil count of more than 0.4 × 10^9^/L.^18^ It is most commonly a benign, transient adverse effect that does not necessitate the discontinuation of clozapine,^18,19^ and if clozapine were previously discontinued because of asymptomatic eosinophilia, clozapine treatment can be rechallenged.^20^ The detailed mechanism of clozapine-induced eosinophilia is unknown, but it has been hypothesized to be an allergic hypersensitivity reaction to clozapine. One of our patients tolerates clozapine treatment now after several commencements, which supports this hypothesis.

Case reports of clozapine-induced eosinophilia associated with end-organ damage such as pleural effusions,^21^ eosinophilic pneumonia,^22^ pancreatitis,^23^ pericarditis,^24^ hepatitis,^25^ and colitis^7^ have been reported. As eosinophilic gastroenteritis is an uncommon and heterogeneous disease, pathology, epidemiology, and predisposing factors are not well characterized, and the diagnostic criteria have some variance.^26,27^ Other conditions where the number of eosinophils may be increased in the gastrointestinal tract, such as parasitic infections, allergic diseases, and inflammatory bowel disease, should be excluded. Definitive diagnosis of eosinophilic colitis requires typical symptoms and increased eosinophils in biopsy specimens from colon. In 2 of our cases, subsequent eosinophilia with diarrhea was observed. With 2 of our patients, the diarrhea symptoms may be related to the peripheral eosinophilia that followed and possible end-organ damage. However, none of the patients had increased number of eosinophils in their colonoscopy biopsies, and peripheral eosinophilia is not a sufficient criterion for diagnosis.^28^ One of our patients had CLCs in stool, which are an eosinophilic breakdown product that are considered to be a marker of eosinophil death and can stay in tissues even for months.^29^ This is, to our knowledge, the first time CLCs are reported during clozapine treatment. One previous case history reported a patient who developed CLCs in stool after allergic reaction to metronidazole treatment for *Clostridium difficile* diarrhea.^30^

Drug reaction with eosinophilia and systemic symptoms (DRESS) syndrome is a severe reaction to a drug causing diverse clinical symptoms, beginning from 2 to 8 weeks from the initiation of the drug.^31^ Several cases of DRESS have been reported in clozapine-treated patients.^32–34^ The pathogenesis of DRESS has been hypothesized to be a complex interaction of accumulation of drug metabolites caused by a genetic deficiency of metabolizing enzymes, drug hypersensitivity associated with specific human leukocyte antigen genotypes, and possible virus-drug interactions.^31^ It can be speculated whether DRESS and benign and transient clozapine-induced eosinophilia share some similarities. Simultaneous development of blood eosinophilia and colitis symptoms may refer to DRESS-like phenomenon. However, DRESS is typically characterized by a longer latency between drug exposure and disease onset; involves skin, liver, and kidneys; and may present with frequent relapses despite the discontinuation of the culprit drug.^35^ Symptoms in our patients relieved, however, dramatically after withdrawal of clozapine.

## STRENGTHS AND LIMITATIONS

We screened for other possible pathogens quite extensively, which can be considered a strength of this report. All the patients except for one underwent gastroscopies and colonoscopies. However, the limitations are that only one of our patients was scoped during active symptoms, and patient 4 declined colonoscopy, and clozapine was discontinued without knowing the exact etiology of his diarrhea.

## CONCLUSIONS

The presented cases together with previous reports support the notion that diarrhea is a rare but clinically relevant adverse effect in clozapine treatment. Charcot-Leyden crystals in stool may be associated with concurrent diarrhea and eosinophilia during clozapine treatment, which is a previously unreported finding. Further investigations could help to understand mechanism behind this adverse effect and to identify possible common risk factors for the development of diarrhea during clozapine treatment.
